# Healthy and Safe Swimming Week — May 18–24, 2015

**Published:** 2015-05-15

May 18–24, 2015, marks the 11th annual Healthy and Safe Swimming Week (formerly known as Recreational Water Illness and Injury Prevention Week). This observance highlights ways in which swimmers, parents, pool owners and operators, beach managers, and public health can maximize the health benefits of water-based physical activity, while minimizing the risk for recreational water–associated illness and injury. More information is available at http://www.cdc.gov/healthywater/observances/hss-week/index.html.

This year’s theme, “Make a Healthy Splash: Share the Fun, Not the Germs,” focuses on a few easy and effective steps swimmers and parents can take to protect themselves and their families and friends from infectious pathogens in pools, waterparks, hot tubs, spas, and water playgrounds. These steps are highlighted in CDC’s new Healthy Swimming brochure, available with other free promotional materials at http://www.cdc.gov/healthywater/swimming/resources/index.html.

CDC also released the 1st Edition of the Model Aquatic Health Code in August 2014 (1), a voluntary guidance document that can help state and local authorities and the aquatics sector make swimming and other water activities healthier and safer. The first Conference for the Model Aquatic Health Code (CMAHC) will be held October 6–7, 2015, in Scottsdale, Arizona, where CMAHC members* can vote on potential MAHC changes. A public health communications toolkit for Healthy and Safe Swimming Week is available at http://www.cdc.gov/healthywater/observances/hss-week/response-tools-public-health.html.

## Footnotes

* More information on how to become a CMAHC member is available at http://www.cmahc.org/membership.php.
